# Analysis of CO_2_ Facilitation Transport Effect through a Hybrid Poly(Allyl Amine) Membrane: Pathways for Further Improvement

**DOI:** 10.3390/membranes10120367

**Published:** 2020-11-25

**Authors:** Bouchra Belaissaoui, Elsa Lasseuguette, Saravanan Janakiram, Liyuan Deng, Maria-Chiara Ferrari

**Affiliations:** 1 LRGP-CNRS, University of Lorraine, ENSIC, 1 rue Grandville, 54001 Nancy, France; 2 School of Engineering, University of Edinburgh, Robert Stevenson Road, Edinburgh EH9 3FB, UK; e.lasseuguette@ed.ac.uk (E.L.); M.Ferrari@ed.ac.uk (M.-C.F.); 3 Department of Chemical Engineering, Norwegian University of Science and Technology (NTNU), NO-7491 Trondheim, Norway; saravanan.janakiram@ntnu.no (S.J.); liyuan.deng@ntnu.no (L.D.)

**Keywords:** facilitated transport, fixed site carrier membrane, polyallylamine-polyvinyl alcohol-graphene oxide membrane, modelling, carbon capture, gas permeation

## Abstract

Numerous studies have been reported on CO_2_ facilitated transport membrane synthesis, but few works have dealt with the interaction between material synthesis and transport modelling aspects for optimization purposes. In this work, a hybrid fixed-site carrier membrane was prepared using polyallylamine with 10 wt% polyvinyl alcohol and 0.2 wt% graphene oxide. The membrane was tested using the feed gases with different relative humidity and at different CO_2_ partial pressures. Selected facilitated transport models reported in the literature were used to fit the experimental data with good agreement. The key dimensionless facilitated transport parameters were obtained from the modelling and data fitting. Based on the values of these parameters, it was shown that the diffusion of the amine-CO_2_ reaction product was the rate-controlling step of the overall CO_2_ transport through the membrane. It was shown theoretically that by decreasing the membrane selective layer thickness below the actual value of 1 µm to a value of 0.1 µm, a CO_2_ permeance as high as 2500 GPU can be attained while maintaining the selectivity at a value of about 19. Furthermore, improving the carrier concentration by a factor of two might shift the performances above the Robeson upper bound. These potential paths for membrane performance improvement have to be confirmed by targeted experimental work.

## 1. Introduction

**Figure 1:**
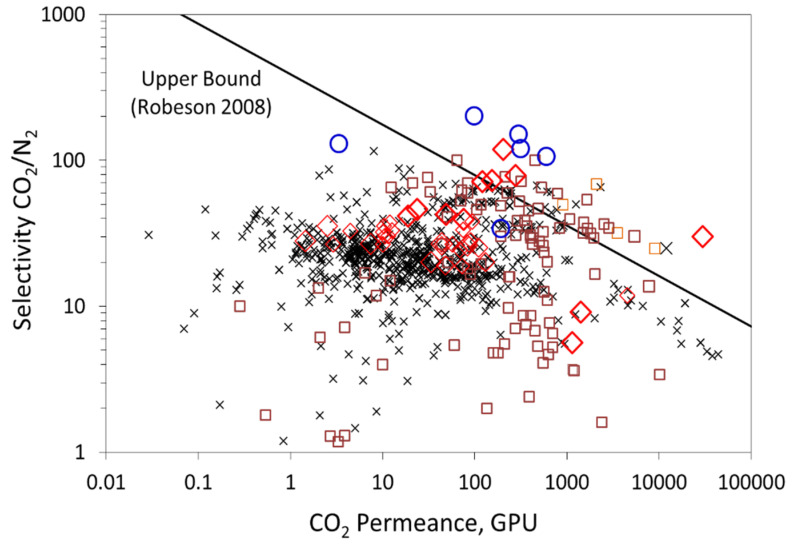
State of the art: trade-off curve showing the CO_2_/N_2_ selectivity (*α*) data for different membrane materials versus CO_2_ permeance for a 1-μm-thick membrane. ✕: polymeric membranes, ☐: inorganic membranes, ◯: facilitated transport membranes,◇: hybrid organic-inorganic membranes. The theoretical trade-off limit, calculated for a strict physical separation mechanism through a dense polymer (i.e., solution-diffusion), is also shown. A membrane thickness of one micron is considered for the upper bound to convert permeability in Barrer to permeance in GPU.

Post-combustion capture (PCC) is an efficient strategy to achieve greenhouse gas emission reductions, as it can be retrofitted to existing power stations or industrial plants and can be integrated into new ones. In the post-combustion framework, flue gases are treated at atmospheric pressure and carbon dioxide is diluted in nitrogen with a typical CO_2_ volume fraction of 5% (natural gas turbine exhaust), 15% (coal combustion power plant), and 30% (steel plant or oxygen enriched air flue gas). As a result, CO_2_ partial pressure in the flue gas is very low, creating a major engineering challenge, especially in terms of energy and membrane surface area requirements for the separation process [1,2]. Many studies have been dedicated to improving existing and already mature technologies (i.e., gas-liquid absorption in amine solvents, cryogenic separation, adsorption). Their success hinges on their ability to lower the cost of CO_2_ capture while still attaining the targets for CO_2_ purity and recovery ratio. Membrane separation processes represent an interesting alternative as a more energy-efficient process with no need for chemicals and no extra source of direct pollution. Commercial gas separation membranes are mainly based on dense polymers, such as polyimide, polysulfone, polycarbonate, polyphenyl oxide, cellulose derivatives, or poly(ethylene oxide) [3,4], and follow the solution-diffusion mechanism. The key shortcoming of solution-diffusion membranes is the trade-off between permeability and selectivity, governed by the Robeson’s upper bound, as shown in Figure 1 [5,6]. The development of membrane materials with high permeability and selectivity is a key challenge for efficient CO_2_ separation. High selectivity is essential to achieve the purity target at low energy cost, and high permeability is required to minimize the membrane surface area and related cost [7,8]. The challenge is then to push the material performances to the right upper side of the Robeson plot.

Among these membranes, facilitated transport membranes (FTMs) have gained much interest in recent years and have shown a promising performance beyond the Robeson upper bound region [3,4,9]. These membranes are based on a selective reversible reaction between the incorporated carrier agents and the target gas component. Facilitated transport membranes for CO_2_ separation most commonly contain amino groups [10,11,12]. Polyvinylamine (PVAm) is one of the most intensively studied fixed-site carrier polymeric membrane materials [13,14].

**Figure 2:**
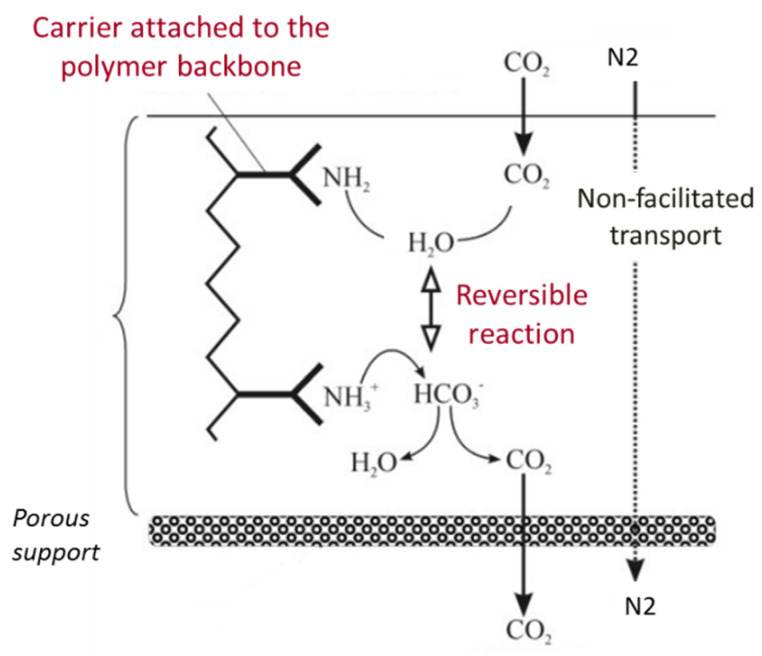
Illustration of the facilitated transport mechanism of fixed-site amine carrier membrane.

In such membrane, the carrier is covalently bonded to the polymer backbone and a CO_2_ molecule reacts with one carrier site in the presence of water, which induces the formation of bicarbonate (HCO_3_^−^) on the feed side interface of the membrane (Figure 2); the complex diffuses along its concentration gradient to the permeate side of the membrane, “hopping” through fixed carrier sites until it reaches the permeate side [15,16]. CO_2_ is released on the permeate side, regenerating the carrier, which can react with another CO_2_ molecule on the feed side. Therefore, a major part of CO_2_ is transported by the carriers inside the membrane, in addition to the physical solution-diffusion mechanism, which other non-reactive gases also follow (Figure 2). A measure of the facilitation effect is the facilitation factor, defined as the ratio of total solute flux with the carrier present to the solute diffusional flux. It represents the contribution of the CO_2_-carrier reaction to the overall transport. Thus, a high facilitation factor corresponds to high selectivity as the flux of the facilitated solute is enhanced in comparison to the diffusion flux of other components in the mixture to be separated.

From the theoretical point of view, many studies have been carried out to calculate and predict the permeation rates and facilitation factors. The general formulation of the facilitated transport of a gas in a membrane can be described mathematically by means of non-linear differential equations expressing a diffusion reaction mass balance of the species involved. The treatment of the nonlinear diffusion reaction (NLDR) problem has been developed considering approximate analytical and/or numerical solutions. Despite the advance in the numerical techniques and computational power, the approximate analytical solutions are still useful because they are more flexible and reliable for extreme cases, such as the chemical equilibrium regime or/and excess carrier. As an example, assuming a large excess of carrier compared to solute is equivalent to assuming that the carrier concentration is constant across the membrane thickness. With this assumption, the NLDR problem describing this process becomes linear and can be solved easily. Several analytical solutions have been proposed to predict the facilitation factor of fixed-site carrier membranes [16,17,18,19,20,21]. In the conditions where these analytical models are applicable, they are accurate despite the simplicity of their mathematical description.

Generally, several studies were reported on membrane synthesis, but few works have dealt with the interaction between material synthesis and transport modelling aspects. In facilitated transport systems, the interaction between both chemistry and chemical engineering is key to understanding the relationship of facilitated transport system properties to separation performances. The performance of a facilitated transport membrane process is dependent upon a number of physical and chemical properties. These properties can be independently measured or retrieved thanks to appropriate analytical transport solutions at suitable operating conditions [22]. Indeed, the interaction of modelling and experimentation is very useful in improving the knowledge base and permits optimization of the facilitation transport mechanism at both the material and process scale [16,23].

For the same combination of carrier and key permeant (here CO_2_), improvement of the membrane performance can possibly be achieved by the modification of the total carrier concentration and the selective layer thickness. Their effect on CO_2_ permeability and selectivity is not trivial [23,24].

On the one hand, decreasing the membrane thickness will not systematically increase the permeance of CO_2_. Indeed, even if the solution-diffusion permeance mechanism of both CO_2_ and N_2_ increases, the transport of CO_2_ by the facilitation mechanism can be reduced significantly, due to reaction limitation (reduced inverse Damkoler number). The effects of the two mechanisms balance, and the facilitated effect (and thus selectivity) can be depleted. On the other hand, the possible positive effect of increasing the carrier concentration has to be weighted by the possible depletion of the reaction complex effective diffusivity. Indeed, the mobility of the complex is a function of the product of both an increasing carrier concentration and reaction complex effective diffusivity, as has been pointed out in the literature [25]. This has to be confirmed by targeted experimental work.

For these reasons, a combined modelling and experimental strategy was used in the present study, in order to show if improvement of the actual membrane performance through chemical and structural modifications is possible and how this could be achieved and evaluated. The results could be used to guide future experimental work. Such a combined analysis is infrequent in the literature.

In this work, first, a hybrid fixed-site carrier membrane using polyallylamine (PAA) with 10 wt% polyvinyl alcohol (PVA) and 0.2wt% graphene oxide was prepared. In order to increase membrane resistance to plasticization and compaction under relevant industrial conditions of CO_2_ capture, hybrid membranes combining the aforementioned polymers and inorganic additives were recently investigated, with the inorganic fillers used as a reinforcing agent in the membrane structure [26,27]. Experimental measurements of CO_2_ and N_2_ fluxes through a hybrid fixed-site carrier membrane, based on poly(allyl amine) matrix, under different operation conditions of the feed relative humidity and CO_2_ upstream partial pressure, are presented and discussed. Second, analytical solutions of the facilitation factor are presented and used to analyze the experimental results and estimate some key facilitation properties. A dedicated parametric analysis was performed to show how the facilitation effect of CO_2_ transport through the studied membrane is affected by the key properties of the membrane, mainly total carrier concentration and membrane selective layer thickness. Indeed, their effect on CO_2_ permeability and selectivity is not obvious. Moreover, the modelling results are compared to actual membrane performances to determine if significant improvements are possible in system performance and how this could be achieved and evaluated. Finally, pathways for membrane chemical and structural modifications are proposed in order to increase the CO_2_ facilitated transport and improve the membrane separation performance.

## 2. Modelling Background

The most common and generalized reaction scheme for the facilitated transport mechanism of CO_2_ across fixed-site amine carrier membranes is expressed as A + C = AC, in which three species co-exist: the key solute (A), here CO_2_, being transported across the membrane; the active chemical carrier (C); and their reaction products or active carrier–solute complex (AC) [26,27].

The solute is transferred from one boundary to the other by two different mechanisms, pure diffusion in an unreacted state and diffusion as a complexed species. Considering a flat-plane geometry with one-dimensional transport (z-direction), Fick’s law diffusion mechanism, and constant diffusion coefficient of the species, the total solute flux can be expressed as:

(1) $$J_{A}^{}=-D_{A}\frac{dC_{A}}{dz}-D_{Ac}\frac{dC_{Ac}}{dz}.$$

The facilitation factor *F* is an evaluation of the impact of the facilitation reaction compared to the pure diffusion mechanism. It is a measure of the increased selectivity resulting from the selective facilitation transport of the key component in a mixture. It is defined as the ratio of the total solute flux of A inside the membrane to the pure solution-diffusion (SD) flux, representing the contribution of the reaction to the overall transport:

(2) $$F=\frac{J_{A}}{J_{A}^{SD}}=\frac{total\;solute\;flux}{\;solute\;solution\;diffusion\;flux}$$

The general formulation of the facilitated transport of a gas in a membrane can be described mathematically by means of differential equations expressing a steady-state nonlinear diffusion reaction (NLDR) problem. The species transport consists of simple diffusion coupled with a single chemical reversible reaction. The forward rate reaction is assumed to depend on both the carrier and solute concentration, while the backward reaction has a linear dependence on the reaction product concentration. Both kinetic constants, *k_f_* and *k_r_*, are considered concentration independent (with *K_eq_* = *k_f_/k_r_*). The mathematical derivation of the mass balance for fixed-site carrier membranes is an analogue to the mobile carrier formulation while an excess of carrier is considered [27]. Indeed, assuming excess carrier, the concentration of the carrier can be considered constant and defined by the reaction equilibrium calculated at the membrane upstream side [18]. Accordingly, the differential equations that describe the steady-state solute transport for fixed-site carrier membranes are:

(3) $$D_{A}\frac{\partial^{2}C_{A}}{\partial z^{2}}=k_{f}\left(C_{A}C_{C}-\frac{1}{K_{eq}}C_{AC}\right)$$

(4) $$D_{AC}\frac{\partial^{2}C_{AC}}{\partial z^{2}}=-k_{f}\left(C_{A}C_{C}-\frac{1}{K_{eq}}C_{AC}\right)$$

With

(5) $$C_{C}^{}=\frac{C_{T}^{}}{1+K_{eq}^{}C_{AO}^{}},$$

Typical boundary conditions consist of the fact that the carrier and the product of the reaction are non-volatile and are constrained to stay confined inside the membrane. A constant source of solute A is considered at one boundary and A is removed continuously from the opposite boundary so that the concentration at that boundary is a constant. Only the solutes can cross the membrane boundaries:

(6) $$at\;z=0,\;C_{A}=C_{A0}\;\frac{\partial C_{C}}{\partial z}=\frac{\partial C_{AC}}{\partial z}=0$$

(7) $$at\;z=L,\;C_{A}=C_{AL}\;\frac{\partial C_{C}}{\partial z}=\frac{\partial C_{AC}}{\partial z}=0$$

In a dimensionless form, the differential equation system becomes:

(8) $$\frac{\partial^{2}C_{A}^{*}}{\partial z^{2}}=\frac{\alpha_{m}K}{\varepsilon }\left(C_{A}^{*}C_{C}^{*}-\frac{1}{K}C_{AC}^{*}\right),$$

(9) $$\frac{\partial^{2}C_{AC}^{*}}{\partial z^{2}}=-\frac{K}{\varepsilon }\left(C_{A}^{*}C_{C}^{*}-\frac{1}{K}C_{AC}^{*}\right),$$

And:

(10) $$C_{C}^{*}=\frac{1}{1+K^{*}}.$$

With the following boundary conditions:

(11) $$at\;z^{*}=0,\;C_{A}^{*}=1\;\frac{\partial C_{AC}^{*}}{\partial z^{*}}=0$$

(12) $$at\;z^{*}=1,\;C_{A}^{*}=\frac{C_{AL}}{C_{A0}}\;\frac{\partial C_{AC}^{*}}{\partial z^{*}}=0$$

The treatment of the nonlinear diffusion reaction (NLDR) problem was developed considering approximate analytical and/or numerical solutions [17,18,22,28]. Many analytical solutions have been proposed to predict the facilitation factor of fixed-site carrier membranes. The solution developed by Smith and Quinn [18] assumes an excess of carrier and zero downstream key permeant partial pressure (here CO_2_). According to this model, the facilitation factor, *F*, is expressed in terms of the key dimensionless facilitated transport parameters, *K*, *ε*, *α*, and *λ*, as the following:

(13) $$F=\frac{1+\frac{\alpha_{m}K}{1+K}}{1+\frac{\alpha_{m}K}{1+K}\left(\frac{tanh\lambda }{\lambda }\right)}$$

In this model, the facilitated factor is expressed as a function of key physicochemical properties, such as the reaction rate constant, chemical equilibrium constant, diffusivities of the chemical species, and membrane thickness. They are combined in a number of dimensionless groupings, having physical significance, which are presented in Table 1.

The key dimensionless number appearing in the equations above are defined in Table 1. These properties of the facilitated transport may be combined in a number of dimensionless groupings, having physical significance. The mobility ratio, *α_m_*, can be defined as the reactive versus the diffusive pathway. It is related to the ratio (*D_AC_*/*D_A_*) of the diffusion coefficient of the A-carrier reaction product and that of free solute *A* inside the membrane. The mobility ratio is also proportional to the initial carrier concentration. *k_D,A_* is the sorption coefficient of solute A in the membranes. *C_AO_* is the feed molar concentration of solute A. *K* is a dimensionless equilibrium constant. *ε* is the inverse of a Damkohler number and is a measure of the characteristic reverse reaction time to the characteristic diffusion time; it serves the same function as a Thiele modulus in catalysis.

It can be seen from the definition of *ε* that the latter is thickness dependent. These dimensionless parameters allow for a simplified evaluation of the performance of particular carrier/solute combinations.

According to Equation (13), *F* is mainly determined by the value of tanh*λ*/*λ*. The value of *λ* decreases with the *K* and *α_m_* values, thus high *K* and *α_m_* values are desired. The maximum F is obtained when *λ* tends to infinity (reaction equilibrium) and thus tanh*λ*/*λ* to zero while F decreases to one when *λ* tends to zero and thus tanh*λ*/*λ* to one (reaction kinetics limitations).

The value of tanh *λ*/*λ* is then a measure of the facilitation effect. Indeed, a simple and quick calculation of this one term can give the range of the facilitation effect factor [17]. Accordingly, one may determine the necessary property modifications to move toward reaction equilibrium by moving the above quantity toward zero and thus to rich high facilitation factors [17]. 

In the limit of the equilibrium reaction, Equation (13) becomes:

(14) $$F=1+\frac{\alpha_{m}K}{1+K}.$$

Generally, the key permeant (here CO_2_) flux through the membrane is expressed as a sum of two contributions: solution diffusion flux of free CO_2_ and flux of the CO_2_-amine complex. Assuming the reaction equilibrium approximation throughout the membrane, the total CO_2_ flux through the membrane film can be calculated as follows [29]:

(15) $$J_{A}=D_{A}\frac{k_{D,A\;}}{e}\left(p_{A}^{\prime }-p_{A}^{\prime \prime }\right)+D_{AC}K_{eq}C_{T}\frac{k_{D,A\;}}{e}\left[\frac{p_{A}^{\prime }}{1+K_{eq}k_{D,A\;}p_{A}^{\prime }}-\frac{p_{A}^{\prime \prime }}{1+K_{eq}k_{D,A\;}p_{A}^{\prime \prime }}\right],$$

where $p_{A}^{\prime }$ and $p_{A}^{\prime \prime }$ are the upstream and downstream partial pressure of A, respectively.

Accordingly, the facilitated factor can be expressed as the following:

(16) $$F=1+\frac{D_{AC}K_{eq}C_{T}}{D_{A}\left(p_{A}^{\prime }-p_{A}^{\prime \prime }\right)}\left[\frac{p_{A}^{\prime }}{1+K_{eq}k_{D,A\;}p_{A}^{\prime }}-\frac{p_{A}^{\prime \prime }}{1+K_{eq}k_{D,A\;}p_{A}^{\prime \prime }}\right]$$

In case of a downstream CO_2_ pressure equal to 0, Equation (16) reduces to Equation (14).

Table 2 summarizes the hypothesis and the facilitation expression of each model, namely: (i) the simplified equilibrium model (Equation (14)); (ii) the equilibrium model expression, which considers a non-zero downstream concentration (Equation (16)); and (iii) the general model expression (Equation (13)). 

## 3. Membrane Fabrication

PAA-PVA-GO membrane was prepared at the Norwegian University of Science and Technology (Group of Prof. L. Deng) in the framework of NanoMEMC^2^ project. It is a composite membrane based on a selective layer composed of a mixture of 0.2 wt% graphene oxide (GO), 89.8 wt% poly(allyl amine) (PAA), and 10 wt% poly(vinyl alcohol) (PVA), coated onto a porous support made of polyvinylidene fluoride (PVDF). The choice of the polymer and nanofiller composition is attributed to the superior performance recorded from previous studies [29]. The use of PVA is to exploit its excellent film-forming capabilities while the use of GO as nanofiller enhances selective gas transport by reorienting polymer chain packing and increasing water distribution in the polymer matrix. Several tests have been conducted in these membranes recently, with studies explaining the role of the individual components present in such hybrid membranes [30]. These membranes have also been validated in industrial testing conditions [31].

Poly(allyl amine) was obtained by purification of poly(allyl amine hydrochloride) (Mw = 120,000 g/mol) (bought from Thermo Fisher Scientific, Stockholm, Sweden) using equivalent potassium hydroxide (KOH) in methanol at room temperature for 24 h. The purified poly(allyl amine) was recovered by centrifuging the supernatant comprising of PAA in methanol. The solution was then dried in a ventilated oven at 60 °C to result in dry polymer, ready to be re-dissolved in deionized water. About 6 wt% solution of PAA in water was obtained by stirring (at 500 rpm) for 24 h. In the case of PVA, a 4 wt% polymer solution in water was prepared by dissolving PVA pellets in deionized water at 80 °C for 4 h under reflux conditions. GO dispersion was obtained as 2 mg mL^−1^ from Graphene-XT, Bologna, Italy.

In order to prepare the casting solution for membrane fabrication, the calculated amount of GO solution was first diluted using water. Drops of both 6 wt% PAA and 4 wt% PVA solutions were then carefully added to the diluted dispersion under stirring conditions to result in a casting solution, which contained 89.8 wt% PAA, 10 wt% PVA, and 0.2 wt% GO. The total solid contents (polymer + GO) in the casting solution were maintained at 2wt%.

**Figure 3:**
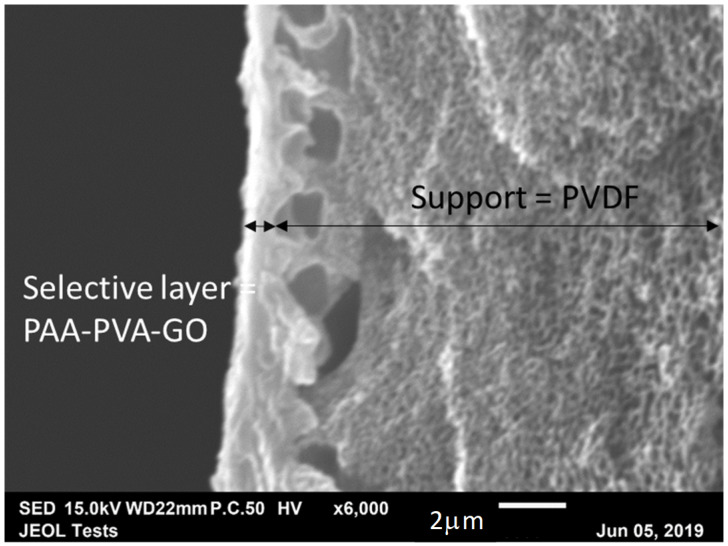
SEM images of a cross-section of polyallylamine (PAA)-poly(vinyl alcohol) (PVA)-graphene oxide (GO) membrane.

The composite membrane was fabricated using the bar coating method mentioned elsewhere [32]. The porous support was washed with tap water at 45 °C for 1 h and DI water for 30 min to remove the pore protection agents. The support was then mounted on to a flat glass plate using aluminum tape. The polymer cast solution was then casted over the support and the membrane was dried at 60 °C in vacuum for 4–5 h. With the bar coating method, defect-free selective layer coating was obtained with the use of FC-72 electronic liquid (3M™ Fluorinert™) as the pore-filling agent. Scanning electron microscopy (Figure 3) revealed that the selective skin thickness was approximately 1 μm. Based on the physical properties of PAA (Mw = 12,000 g/mol, density = 1.02 g/cm^3^), it was possible to determine a total carrier concentration in the membrane of 1.61 × 10^−2^ mol·cm^−3^.

## 4. Experiments: Results and Discussion

Gas permeability measurements were performed with a mixed gas–continuous flow permeation cell designed at the University of Edinburgh; details on the permeation cell and the experimental procedure can be found in a previous article [33]. A flat circular membrane with an area of 2.835 cm^2^ was used for the experiments.

The operating conditions used for our project are summarized in Table 3. The downstream pressure, feed and sweep flow rates, and sweep gas relative humidity were set constant, whereas the humidity content and the CO_2_ partial pressure in the feed side were varied from 20% to 90% and from 0.15 to 3 bar, respectively.

The permeability ${℘}_{i}$ and the permeance $PM_{i}$ were calculated from the experimental data of the measured fluxes, *J_i_*_,_ as follows:

(17) $$PM_{i,\;i=CO2,N2}=\frac{J_{i}\;}{S\;\left(p_{i}^{\prime }-p_{i}^{\prime \prime }\right)}=\frac{{℘}_{i,\;i=CO2,N2}}{e},$$

where *e* is the thickness of the membrane (cm), S is the membrane surface area, *J_i_* is the gas permeate flowrate of component j (cm^3^ (STP)/s), A is the effective membrane area (cm^2^), and $p_{i}^{\prime }$ and $p_{i}^{\prime \prime }$ are the partial pressure of the gas in the feed and permeate stream, respectively (cmHg). 

The CO_2_/N_2_ ideal selectivity $\alpha_{CO2/N2}$ between two gas species was calculated as the ratio of the two permeabilities:

(18) $$\alpha_{CO2/N2}=\frac{{℘}_{CO2}\;}{{℘}_{N2}}.$$

The common unit of permeability is Barrer, which corresponds to 10^−10^ cm^3^ (STP) cm cm^−2^ s^−1^ cmHg^−1^. The permeance is defined as the ratio of permeability to the selective layer thickness. Its common unit is GPU (gas permeation unit), which corresponds to 10^−6^ cm^3^ (STP) cm^−2^ s^−1^ cmHg^−1^.

### 4.1. Effect of Feed Relative Humidity 

A large relative humidity is required for the facilitated transport reaction mechanism to operate in the polymer [34]. Moreover, the impact of humidity on the solution diffusion mechanism can also be important. Indeed, a high water content is likely to swell rubbery membranes, increasing the polymeric chain mobility and as a result inducing increased gas permeabilities and possible loss in CO_2_/N_2_ selectivity. Moreover, a loss of CO_2_ permeability can be observed in glassy polymer due to the competitive sorption between water and CO_2_ and the free volume reduction effect [34,35,36,37].

**Figure 4:**
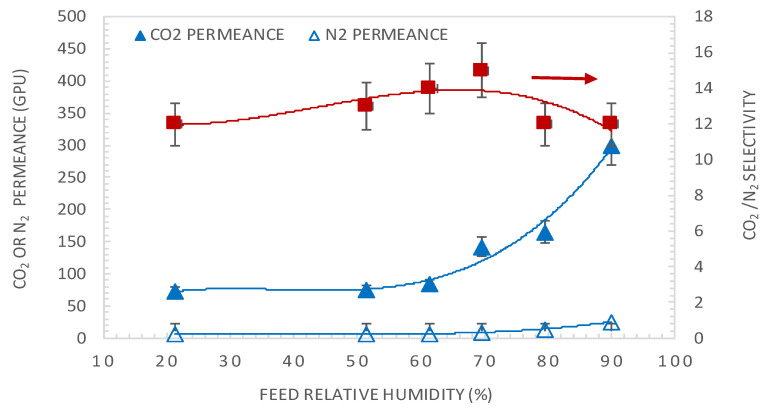
The effect of feed relative humidity on the CO_2_ and N_2_ permeances and CO_2_/N_2_ membrane selectivity, at a feed CO_2_ partial pressure of 1.5 bar and temperature of 50 °C. Lines are to guide the eye.

Figure 4 shows the effect of variation of the feed relative humidity (RH_f_) on the CO_2_ and N_2_ permeances and CO_2_/N_2_ membrane selectivity. The RH_f_ varied from 20% to 90% with a constant sweep gas relative humidity of 50% and a temperature of 50 °C. It can be seen that both CO_2_ and N_2_ permeances increase with RH_f_, with CO_2_ permeance increasing exponentially. Indeed, an increase in RH leads to a higher water content in the membrane matrix, which enhances the reaction between CO_2_ and amine carriers and the mobilities of the reaction products but also unreacted CO_2_ and N_2_ [27,34]. Regarding the CO_2_/N_2_ selectivity, it shows a non-monotonic trend as it shows a slight maximum at a relative humidity of 70%. A selectivity drop at a high RH might be associated with enhanced penetration of non-reacting gas molecules relative to the contribution of facilitated transport due to the swelling of the membrane at high water content. The same trend was also observed by Sandru et al. [14] using PVAm/PPO for CO_2_/N_2_ separation at a feed pressure of 2.2 bar, 10% CO_2_–90% N_2_ feed at 25 °C. They showed that with increasing humidity content, the CO_2_/N_2_ selectivity increased to the maximum at 65% feed RH and then decreased. The same trend was also observed by Ansaloni et al. [27] using amino-functionalized multi-walled carbon nanotubes under high pressures (15–28 bar) and high temperatures (103–121 °C). They showed that the CO_2_/CH_4_ and CO_2_/H_2_ selectivity increased with feed RH until a maximum at 72% RH and then decreased. This trend has already been reported in the literature and can be explained by the occurrence of important water-induced swelling of the membrane at a high relative humidity content in the gas, which impaired the barrier property of the membrane toward N_2_ [27].

### 4.2. Effect of CO_2_ Partial Pressure

**Figure 5:**
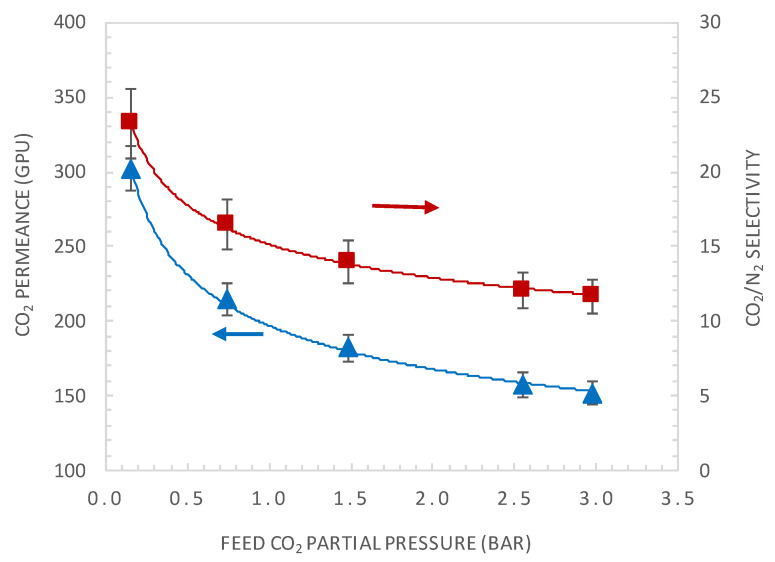
The effect of CO_2_ upstream partial pressure on the CO_2_ permeance and CO_2_/N_2_ selectivity, at a feed relative humidity of 85% and temperature of 40 °C.

A general characteristic of facilitated transport membrane is that the CO_2_ permeance is pressure dependent, as the carrier is saturated under high CO_2_ partial pressure, resulting in decreasing CO_2_ selectivity over other gases. Figure 5 shows the effect of varying the CO_2_ upstream partial pressure on the CO_2_ permeance and CO_2_/N_2_ membrane selectivity. The CO_2_ upstream partial pressure varied from 0.15 to 3 bar with a feed relative humidity of about 85% and a temperature of 40 °C. The permeance of N_2_ was found to be independent from the feed pressure and has a value of 13 GPU under the investigated operating conditions. As illustrated in this figure, CO_2_ permeance and selectivity decrease with the feed pressure and then reach a constant value. This can be explained by the carrier saturation phenomenon [11,38]. For CO_2_ partial pressure of 0.15 bar, the actual membrane CO_2_ permeance is of 302 GPU and a CO_2_/N_2_ selectivity of 23.2.

From the above results, it can be seen that the best performances of the membrane correspond to a permeance of 300 GPU and CO_2_/N_2_ selectivity of 24. These performances are located below the upper-bound Robeson plot shown in Figure 2. The objective of the present work was to show through a dedicated combined modelling and experimental strategy if further improvement of the actual membrane performance is possible and how this could be achieved and evaluated. The results could be used to guide future experimental work.

## 5. Simulations: Results and Discussion 

### 5.1. Retrieval of Key Facilitation Parameters

The physical and chemical properties of the facilitation factor can be independently measured or estimated. The analytical expression, being simple, offers the possibility to estimate some key system properties based on transport measurements and simple fitting techniques. Indeed, the analytical solution presented above can provide the basis for estimating the carrier gas complex diffusion coefficient and the reaction equilibrium constant. Assuming reaction equilibrium and a negligible CO_2_ downstream concentration, the analytical solution of Smith and Quinn [18], Equation (14), can be rearranged as:

(19) $${\left(F-1\right)}^{-1}=E=\alpha_{m}{}^{-1}+{\left(\alpha_{m}K\right)}^{-1}=\left(\frac{D_{A}k_{D,A\;}}{D_{AC}C_{T}}\right)p_{A}^{\prime }+\left(\frac{D_{A}}{D_{AC}C_{T}}\right)K_{eq}{}_{}$$

Thus, by varying the solute feed concentration and measuring the permeant flux, one can calculate the facilitation factor, *F*, and make a plot of *E* = (*F* − l)^−1^ versus the solute upstream partial pressure. A straight-line relationship (as in Equation (19)) implies reaction equilibrium, i.e., diffusion-limited system (tanh *λ*/*λ* ~ 0). Deviation from a straight line indicates reaction limitations (tanh *λ*/*λ* ≠ 0). Considering the straight-line part of the curve, at a low CO_2_ upstream concentration, the slope and intercept can be used to determine the complex diffusion coefficient *D_AC_* and the reaction equilibrium constant *K_eq_*, respectively [19,37,38].

The total carrier concentration, *C_T_*, for the tested membrane was calculated to be 1.61 × 10^−2^ mol·cm^−3^. This value is in accordance with the literature and among the highest values [39,40]. The CO_2_ permeance corresponding to the solution-diffusion mechanism, $PM_{CO2}^{SD}$, through the membrane was set at 145 GPU (+/−4), determined from experimental data under high permeant partial pressure corresponding to carrier saturation, conditions where CO_2_ flux increase is only due to the pure solution –diffusion mechanism. Indeed, we suppose that saturation occurs at 3 bars, but this is an approximation, as saturation could occur at higher pressure; indeed, a starting pseudo-plateau can be observed for pressures above 2.5 bar. The CO_2_ diffusion coefficient was set at a value of 1 × 10^−6^ cm^2^·s^−1^, which is in the range reported in the literature with respect to the order of magnitude of amine-functionalized carrier membranes [28,41,42,43,44].

Starting from the value of CO_2_ permeance in the SD mechanism and the value of the CO_2_ diffusion coefficient, the CO_2_ sorption coefficient, *k_d_*, can be calculated according to the following equation:

(20) $${℘}_{CO2}^{SD}=k_{D,CO2}D_{CO2}=PM_{CO2}^{SD}*e_{}.$$

For our system, we found a value of *k_d_* = 4.84 × 10^−5^ moL·cm^−3^·bar^−1^, which is comparable to values from the literature for amorphous polymers [45].

Thanks to the measurement of the CO_2_ permeabilities, it is possible to calculate an experimental facilitation factor according to the following equation:

(21) $$F_{exp}=\frac{PM_{CO2}\;}{PM_{CO2}^{SD}}.$$

**Figure 6:**
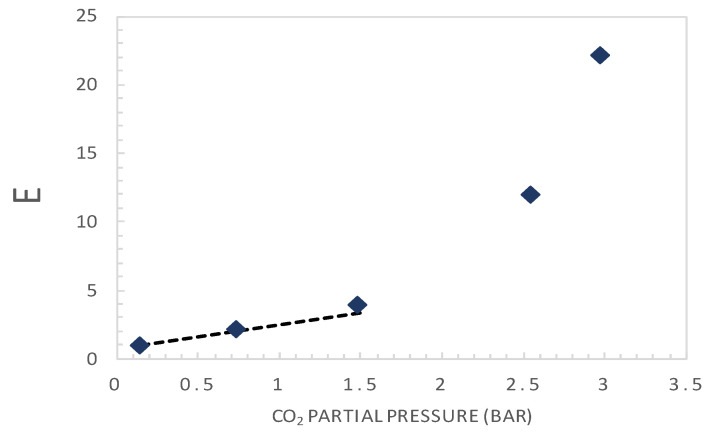
Plot of E as a function of the upstream CO_2_ partial pressure. The dashed line corresponds to a straight-line fitting curve at very low CO_2_ partial pressures (<1.5 bar), according to Equation (19).

The plot of *E* = (*F* − l)^−1^ versus upstream CO_2_ partial pressure is shown in Figure 6.

Applying the analysis exposed above (Equation (7)), which is valid at very low CO_2_ partial pressures, a straight-line fitting curve in this range (<1.5 bar) is plotted and added in Figure 6. Thus, the slope and the intercept of this line are used to estimate the complex diffusion coefficient and the reaction equilibrium constant, respectively.

The retrieved value of D_AC_ is found to be equal to 1.7 × 10^−9^ cm^2^·s^−1^, which is about one order of magnitude lower than that reported in the literature for CO_2_-amine complex. In an ion exchange membrane with ethylene diamine as a carrier [41], a value of about 9.7 × 10^−9^ cm^2^·s^−1^ was estimated. Cussler [15] showed that for fixed-site carrier membrane or “chained carrier” membrane, the order of magnitude of the apparent diffusion coefficient of the reaction complex is 10^−8^ cm^2^·s^−1^. This can explain the low permeance value even if the value of C_T_ is relatively high compared to typical values from the literature. The retrieved reaction equilibrium constant K*_eq_* is 5.2 × 10^4^ cm^3^·moL^−1^. It is found to be in accordance with typical values for the amine−CO_2_ reaction from the literature [41,46]. The value of the reverse reaction constant, *k_r_*, was set at 110 s^−1^, similar to typical values from the literature for the CO_2_–amine reaction [28,47,48].

### 5.2. Comparison of Experimental and Simulation Results

Table 4 summarizes the properties of the membrane used for the modelling analysis.

From the predicted facilitation factor, the permeance of CO_2_ and CO_2_/N_2_ selectivity is calculated, respectively, as follows:

(22) $$PM_{CO2}^{}=F\;\times PM_{CO2}^{SD}$$

(23) $$\alpha =\frac{PM_{CO2}\;}{PM_{N2}^{}}.$$

**Figure 7:**
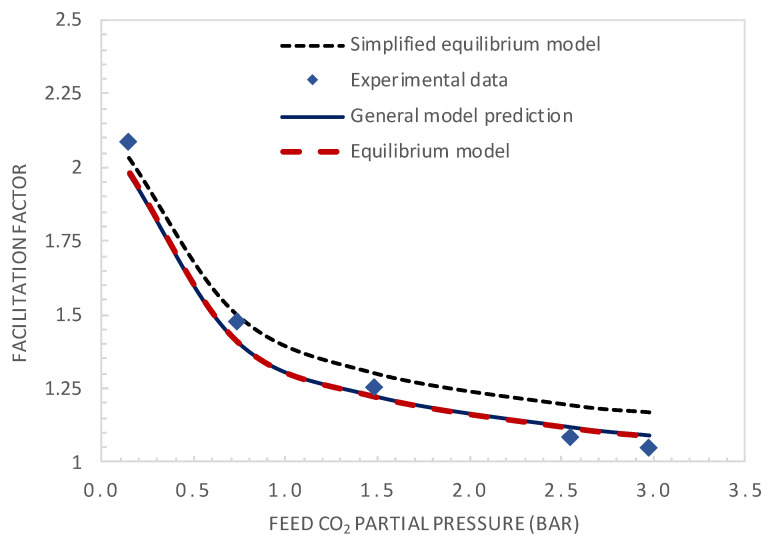
Comparison of the model predictions and experimental values of the facilitation factor.

**Figure 8:**
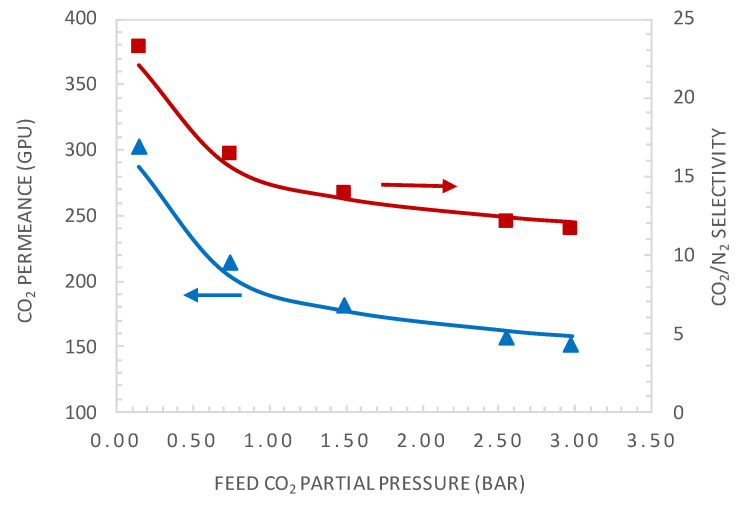
Comparison of CO_2_ permeance and CO_2_/N_2_ selectivity between the general model simulation and experimental values. Simulations are shown in a continuous line.

The experimental and modelling results are compared in terms of the facilitation factor, CO_2_ permeance, and CO_2_/N_2_ selectivity as a function of the CO_2_ upstream partial pressure in Figure 7 and Figure 8, respectively.

The simplified equilibrium model, which assumes reaction equilibrium and negligible CO_2_ permeate pressure (Equation (2)), shows good agreement with the experimental results at low feed CO_2_ partial pressure, as expected. However, it diverges for higher values of the feed CO_2_ partial pressure. Both the equilibrium model and general model show good agreement with the experimental results in the whole investigated CO_2_ feed pressure range, with a maximum deviation between the experimental and predicted values below 5%. Figure 8 shows the comparison of CO_2_ permeance and CO_2_/N_2_ selectivity between the general model prediction (Equation (13)) and experimental values. These results again demonstrate the good fitting ability of the general model.

For the sake of analysis, the dimensionless facilitated transport parameters for the actual membrane were calculated for two limits of the investigated range of upstream CO_2_ partial pressure: P_CO2_ = 0.15 bar and 3 bar. The results are given in Table 5.

The value of *tanh λ/λ*, in Equation (13), is a measure of solute facilitated transport. In the operating conditions of the experiments, the values of *tanh λ/λ* were all lower than 3 × 10^−3^, indicating that the system is operating near the reaction equilibrium regime or diffusional limitation regime. This indicates that, under the actual operating conditions, the facilitation factor is already at its maximum for the actual membrane composition and selective layer thickness. For the actual membrane system, the values of *K* calculated for the two limits of the upstream CO_2_ partial pressure lie globally within the optimal values range [1.5–10], predicted by the substantial numerical parametric analysis of Kemena et al [24,28,38].

The calculated *ε* was below 0.01, indicating again that compared to the reaction rate, the diffusion rate of the reaction complex was the rate-controlling step of the overall CO_2_ transport across the membrane. This also means that there might be room for membrane separation performance improvement by modifying the value of *ε* and the mobility ratio *α_m_*. These modifications can be examined by analyzing the effect of the membrane selective layer thickness and the initial carrier concentration in the membrane, *C_T_*, on membrane performances. 

### 5.3. Parametric Analysis

For the same combination of carrier and key permeant (here CO_2_), improvement of the membrane performance can possibly be achieved by the modification of the total carrier concentration, C_T_, the selective layer thickness, δ, and/or the operating parameters, such as temperature (T), CO_2_ partial pressure (p_CO2_), and relative humidity (RH). The membrane selective layer thickness and carrier concentration are two key characteristics that can be modified in order to improve performances, but their effects on CO_2_ permeability and selectivity are not trivial [22,24]. In this section, a parametric analysis is achieved in order to show if improvement of the actual membrane performance is theoretically possible. The effect of the membrane selective layer thickness and total carrier concentration was evaluated through a parametric analysis, using the general model solution (Equation (13)), presented in the previous section. The membrane selective layer thickness and total carrier concentration were varied around their actual values of 1 µm and 1.61 × 10^−2^moL·cm^−3^, respectively.

The permeability of N_2_ as well as the SD CO_2_ permeability were kept constant at the experimentally measured values of 13 and 145 Barrer, respectively, regardless of the total carrier concentration inside the membrane and CO_2_ upstream partial pressure. The SD permeance for a given membrane thickness was then calculated according to Equation (20). From the predicted values of the facilitated factor, the permeance of CO_2_ for a given membrane thickness was calculated according to Equation (22).

#### 5.3.1. Effect of Membrane Thickness

**Figure 9:**
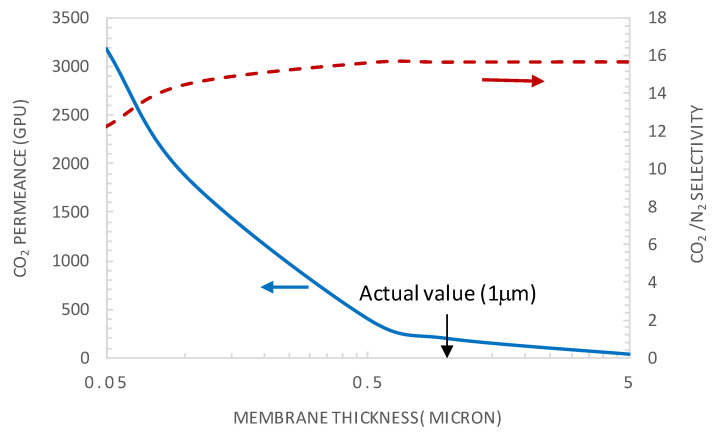
CO_2_ permeance and CO_2_/N_2_ selectivity as a function of the selective layer membrane thickness (general model simulation results). p’_CO2_ = 0.75 bar, CT = CT_0_ = 1.61 × 10^−2^ moL·cm^−3^.

The analytical expression of the facilitation factor clearly indicates, from the definition of *ε*, the thickness dependence of the facilitation factor. Figure 9 shows the variation of the CO_2_ permeance and CO_2_/N_2_ selectivity with the membrane thickness for a CO_2_ partial pressure of 0.75 bar. Indeed, considering that the CO_2_ concentration in the feed gas car varies depending on the emission sources, from 5% (natural gas turbine exhaust) to 30% (steel plant or oxygen enriched air flue gas), and that the flue gas can be pressurized to about 4 bar, a CO_2_ partial pressure of 0.75 bar was taken as an average value.

The selective layer thickness varied in the range 0.05–5 μm. The results in Figure 9 show that the permeance decreases monotonically with increasing membrane thickness. This trend can be explained by the fact that the mass flux through a membrane is inversely proportional to the selective layer thickness. On the contrary, the selectivity shows an increasing trend with an increasing selective layer thickness. Actually, the species diffusion becomes more limiting relative to the reaction, translating into decreased *ε* values, as the latter is proportional to the inverse of the square of the membrane thickness. Consequently, the facilitation effect and CO_2_/N_2_ selectivity increase with an increased selective layer thickness.

Moreover, from Figure 9, it is clear that there is no benefit from increasing the membrane thickness above the actual value of 1 µm, as CO_2_ permeance will deplete without any substantial positive effect on membrane selectivity. Decreasing the membrane selective layer thickness from the actual value to a value around 0.1 µm permits a significant increase of the CO_2_ permeance without a significant effect on membrane selectivity. As an example, for a membrane thickness of 0.1 µm and CO_2_ upstream partial pressure of 0.75 bar, a CO_2_ permeance of 1880 GPU can be reached while maintaining the selectivity at a value of about 14.47. These performances are already located on the upper bound of the Robeson plot. It is found that the actual value of ε is of about 10^−3^, indicating that the system is under a diffusional limitation regime and experiences the maximum facilitation factor and membrane selectivity [22]. This explains why an increase in membrane thickness above the actual value has no benefit on membrane selectivity as shown in Figure 9.

**Figure 10:**
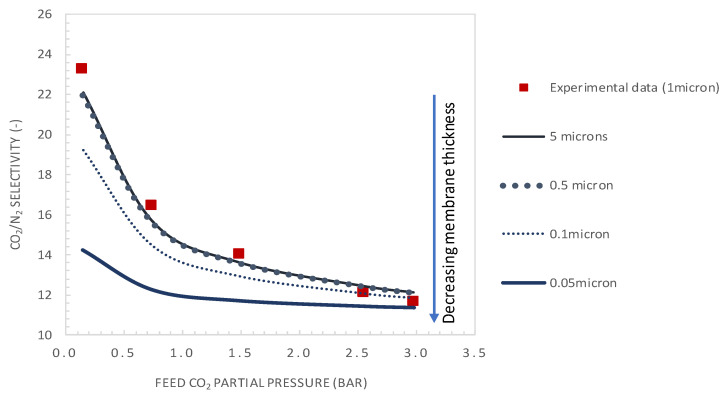
CO_2_/N_2_ selectivity as a function of the feed CO_2_ partial pressure. Results are given for different values of selective layer membrane thickness (general model simulation results). CT = CT_0_ = 1.61 × 10^−2^ mol·cm^−3^.

**Figure 11:**
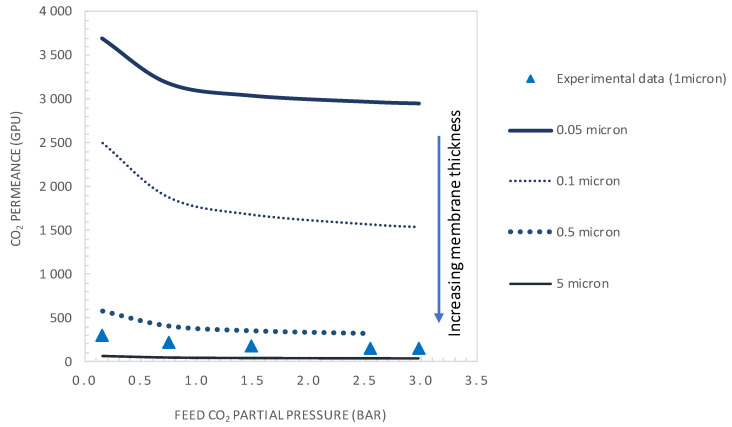
CO_2_ permeance as a function of the feed CO_2_ partial pressure. Results are given for different values of selective layer membrane thickness (general model simulation results). CT = CT_0_ = 1.61 × 10^−2^ mol·cm^−3.^

Figure 10 and Figure 11 show the variation of CO_2_/N_2_ selectivity and CO_2_ permeance as a function of the CO_2_ partial pressure, respectively, for different values of membrane thickness. First, it can be seen that the increase of the membrane thickness above the actual value of 1 µm results in an insignificant improvement of the membrane selectivity (see Figure 10). Furthermore, decreasing the membrane thickness below the actual value to 0.1 µm has a negligible effect on the membrane selectivity and a positive effect on the CO_2_ permeance (see Figure 11). Moreover, the calculated values of tanh *λ*/*λ* are below 0.1, indicating that the system is near reaction equilibrium and thus near the maximum facilitation factor, explaining the negligible effect of increasing the selective layer thickness on membrane selectivity. As an example, for a membrane thickness of 0.1 µm and CO_2_ upstream partial pressure of 0.15 bar, a CO_2_ permeance as high as 2500 GPU can be attained while maintaining the selectivity at a value of about 19. These performances are interestingly above the Robeson upper bound for CO_2_/N_2_ separation. These results highlight the thickness dependence characteristic of the facilitation factor, which has rarely been reported in the literature [10,27]. Generally, the facilitation factor does not directly correspond to absolute permeate flux or capacity. Increasing the membrane thickness (i.e., decreasing *ε*) will continuously increase F toward its maximum value. However, the permeate solution diffusion flux (the denominator in F) decreases as the membrane thickness increases. Moreover, the maximal facilitation factor will give the reader a measure of the best selectivity obtainable for a given set of operating conditions, and this has to be weighed along with the permeate flux obtained when designing such a system [24]. Consequently, in order to increase the selectivity of the investigated membrane system, the modifications could be achieved by increasing the mobility ratio through increased carrier solubility or/and amine-carrier complex diffusivity. The effect of the total carrier concentration modification on the membrane performances is analyzed and discussed in the next section.

#### 5.3.2. Effect of Carrier Concentration

**Figure 12:**
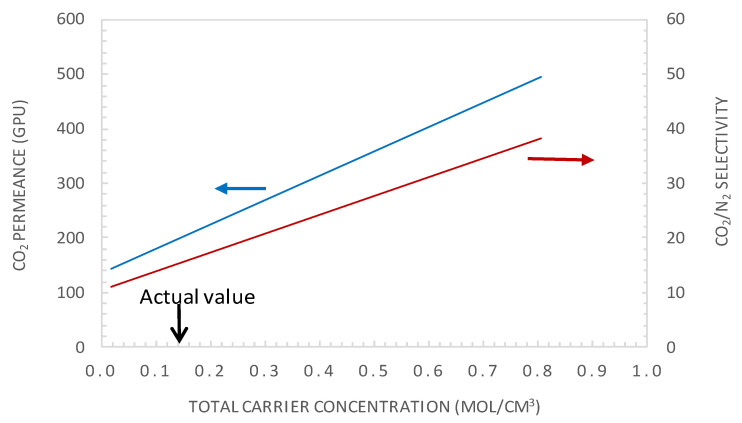
CO_2_ permeance and CO_2_/N_2_ selectivity as the total carrier concentration (general model simulation results). p’_CO2_ = 0.74 bar, e = 1 μm.

The facilitation factor is a function of the mobility ratio *α*_m_, which depends on the ratio (D_AC_/D_A_) of the diffusion coefficient of the CO_2_-amine reaction product to that of CO_2_ inside the membrane and on the initial carrier concentration, C_T_, for a given CO_2_ upstream partial pressure. Figure 12 shows the variation of CO_2_ permeance and CO_2_/N_2_ selectivity with the total carrier concentration for a CO_2_ partial pressure of p_CO2_ = 0.75 bar. The carrier concentration was varied around the actual value of 1.61 × 10^−2^ mol·cm^−3^, noted as C_T0_ in Figure 12. The permeance and selectivity were found to increase linearly with an increasing total carrier concentration. This trend can be explained by the fact that an increase in the mobility ratio, *α*_m_, with the total carrier concentration induces an increase in CO_2_ facilitation transport. Many authors have shown experimentally that the facilitated flux increases linearly as a function of the carrier concentration through different FTMs [41,49,50]. The concentration of carriers in the membrane matrix can be increased by the addition of mobile carriers [30].

**Figure 13:**
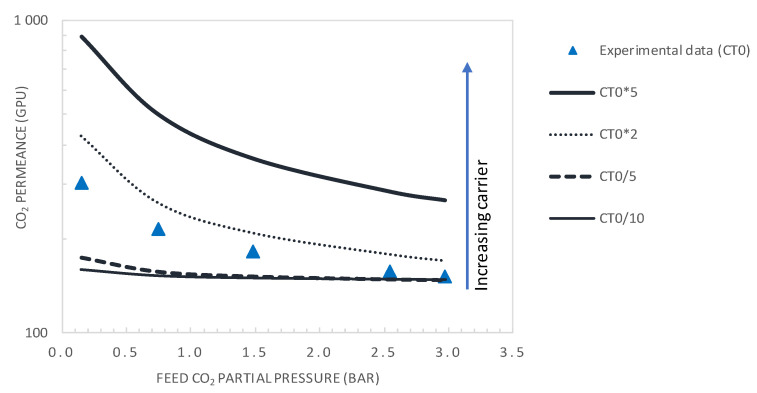
CO_2_ permeance as a function of the feed CO_2_ partial pressure (general model simulation results). Results are given for different values of the total carrier concentration around the actual value of CT_0_ = 1.61 × 10^−2^ mol·cm^−3^.

**Figure 14:**
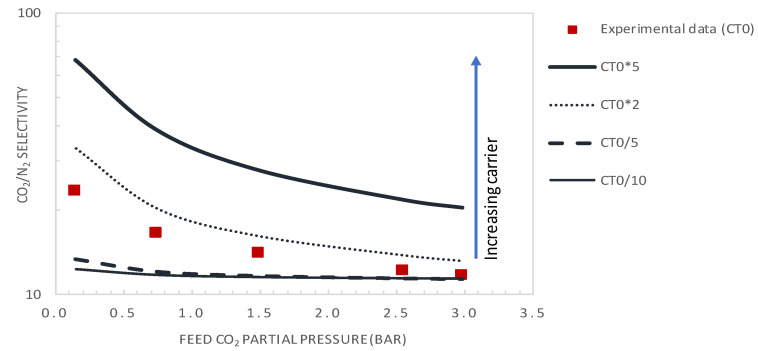
CO_2_/N_2_ selectivity as a function of the feed CO_2_ partial pressure (general model simulation results). Results are given for different values of the total carrier concentration around the actual value of CT_0_ = 1.61 × 10^−2^ mol·cm^−3^.

Figure 13 and Figure 14 show the evolution of the CO_2_/N_2_ selectivity and CO_2_ permeance as a function of the upstream CO_2_ partial pressure, respectively, for different values of the initial carrier concentration. As expected, the results indicate that increasing the C_T_ significantly increases the CO_2_ permeance and membrane selectivity. As an example, for a CO_2_ partial pressure of 0.15 bar, increasing the carrier concentration by a factor of 2 or 5 will significantly increase the membrane performance from its actual performance (*PM*_CO2_ = 302 GPU and *α*_CO2/N2_ = 23.2), to *PM*_CO2_ = 429GPU and *α*_CO2/N2_ = 33 or *PM*_CO2_ = 891 GPU and *α*_CO2/N2_ = 69, respectively. These performances are above the Robeson upper bound of the CO_2_/N_2_ pair.

It is expected that decreasing the selective layer thickness to 0.1 μm together with doubling of the total carrier concentration will shift the membrane performance far above the Robeson upper bound for the CO_2_/N_2_ pair. However, it is worth noticing that the product C_T_*D_AC_ affects the mobility ratio, *α*_m_, and thus the value of the facilitation effect. An increase in C_T_ could be offset by a decrease in D_AC_, subsequent to the change in membrane morphology, as pointed out in the theoretical analysis of Noble [25]. Such a decrease in D_AC_ with carrier loading has been reported in the literature for O_2_ transport by Tsuchida and co-workers [25]. Moreover, on the one hand, an increase of the carrier concentration increases CO_2_-amine complex formation. On the other hand, the salting-out effect might occur at a high carrier concentration. Under these conditions, the formed ionic species (e.g., carbamate, bicarbonate or zwitterion) tend to surround the carrier and the polymeric chain, making it difficult for CO_2_ molecules to access the carrier, depleting CO_2_ solubility and reaction complex diffusion through the membrane [10]. The result of the two opposite effects indicates that an optimal carrier concentration exists. The occurrence of an optimal amine concentration has also been observed experimentally for CO_2_ facilitated transport [51,52,53].

## 6. Conclusions

Based on combined transport measurement and modelling methods, the key membrane system properties were retrieved, and pathways for membrane performance enhancement through chemical and structural modifications (i.e., membrane selective layer thickness and total carrier concentration) were proposed. Experimental measurement of CO_2_ and N_2_ fluxes through a PAA-PVA-GO hybrid fixed-site carrier membrane under different operation conditions was performed. The effects of the humidity content and the CO_2_ partial pressure were investigated. The values of the CO_2_-amine reaction product diffusivity and the reaction equilibrium constant were found to be equal to 1.7 × 10^−9^ cm^2^·s^−1^ and 5.2 × 10^4^ cm^3^·mol^−1^, respectively. For a CO_2_ partial pressure of 0.15 bar, the actual membrane CO_2_ permeance was 302 GPU and CO_2_/N_2_ selectivity was 23.2, performances that were below the well-known Robeson upper bound. The comparison of experimental results and the analytical model predictions showed very good agreement. The major conclusions can be summarized as follows:

1. It was demonstrated that the current system operated near the reaction equilibrium regime (i.e., diffusion limitation), maximizing the facilitated transport of CO_2_. This indicated that, under the investigated operating conditions, the membrane thickness was already at its optimal value maximizing the facilitation factor.
2. Increasing the membrane selectivity of the actual membrane by increasing the mobility ratio through increasing the carrier concentration and amine-CO_2_ complex diffusivity is key to improving the membrane performances.

Furthermore, a parametric analysis regarding the membrane thickness and total carrier concentration was also performed. The main results were:

1. It was shown that after decreasing the membrane selective layer thickness below the actual value of 1 µm to a value of 0.1 µm and CO_2_ upstream partial pressure of 0.15 bar, a CO_2_ permeance as high as 2500 GPU can be attained while maintaining the selectivity at a value of about 19.
2. Moreover, increasing the carrier solubility by a factor of two permitted the attainment of a CO_2_ permeance of 429 GPU and CO_2_/N_2_ selectivity of 33, performances that are above the Robeson upper bound of the CO_2_/N_2_ pair.

Moreover, it is expected that decreasing the selective layer thickness to 0.1 µm together with doubling of the total carrier concentration will theoretically shift the membrane performance far above the Robeson upper bound for the CO_2_/N_2_ pair. However, this potential path for membrane performance improvement has to be weighted by the possible depletion in the reaction complex effective diffusivity, as pointed out in the literature. Finally, it is important to emphasize that the analysis set forth in this paper provides some guidance for membrane performance enhancement through chemical and structural modifications. These potential improvement pathways have to be confirmed by targeted experimental work.

## Figures and Tables

**Table 1:** Definition of the key dimensionless number in facilitation transport.

Dimensionless Numbers	Definition	Expression
*ε*	Inverse Damkohler number, ratio of the characteristic reverse reaction to diffusion time	$\varepsilon =\frac{D\begin{array}{c} AC \end{array}}{k_{r}\;e^{2}}$
*α_m_*	Mobility ratio of mobility of carrier to mobility of solute	$\alpha_{m}=\frac{D\begin{array}{c} AC \end{array}C_{T}}{D_{A}\;C_{A0}}$, with $C_{A0}=k_{D,A}p_{A}^{\prime }$
*K*	Dimensionless reaction equilibrium constant	$K=\frac{k_{f}C\begin{array}{c} A0 \end{array}}{k_{r}\;}=k_{eq}C_{A0}$
*λ*	Measure of the facilitation factor	$\lambda =\frac{1}{2}\sqrt{\frac{1+\left(\alpha +1\right)K}{\varepsilon \left(1+K\right)}}\;$

**Table 2:** Summary of the hypothesis and the facilitation expression of each model.

Model Hypothesis		Model Name	Model Equation (*F* Expression)
Chemical equilibrium (tanh*λ*/*λ* ~ 0)	Zero downstream CO_2_ concentration ($p_{CO_{2}}^{\prime \prime }$ = 0) (very dilute CO_2_ gas)	Simplified equilibrium model	**Equation (14)**
	Non-zero downstream CO_2_ concentration	Equilibrium model	**Equation (16)**
Non-chemical equilibrium (reaction limitation)	Zero downstream CO_2_ concentration	General model	**Equation (13)**

**Table 3:** Operating conditions.

Operating Conditions	Value
Temperature °C	40 and 50
Feed composition	10−100% CO_2_/N_2_
Sweep gas composition	Pure He
Feed pressure (bar)	1–3
CO_2_ partial pressure, P_CO2_ (bar)	0.15–3
Sweep pressure (bar)	1
Feed Relative humidity, RH_f_ (%)	20–90
Sweep Relative humidity, RH_s_ (%)	50
Feed flow rate, Q_F_ (Ncm^3^/min)	150
Sweep flow rate, Q_s_ (Ncm^3^/min)	10

**Table 4:** Permeation properties used for the modelling analysis.

*D*_*CO*2_ (cm^2^·s^−1^)	*k_d_*,_*CO*2_ (mol·cm^−3^·bar^−1^)	*D_AC_* (cm^2^·s^−1^)	*K_eq_* (cm^3^·mol^−1^)	*K_r_* (s^−1^)	*SDCO*_2_ Permeability (Barrer)	*SDCO*_2_ Permeance (GPU), * e = 1 µm	N_2_ Permeability (Barrer)	N_2_ Permeance (GPU) * e = 1 µm
1 × 10^−6^	4.84 × 10^−5^	1.7 × 10^−9^	5.20 × 10^4^	110	145	145	13	13

* Relative to dimensionless.

**Table 5:** Calculated dimensionless facilitated transport parameters, P_CO2_ = 0.15 and 3 bar.

P_CO2_ (bar)	*α_m_*	*ε*	*K*	*tanhλ/λ*
0.15	3.78	1.54 × 10^−3^	0.377	1.52 × 10^−3^
3	0.91	1.54 × 10^−3^	7.49	2.64 × 10^−3^

## Nomenclature

*D*	Diffusion coefficient (cm^−2^∙s^−1^)
*e*	Membrane selective layer thickness (cm)
*F*	Facilitation factor (dimensionless)
*RH*	Relative humidity (dimensionless)
*K_eq_*	Reaction equilibrium constant (cm^3^·moL^−1^)
*℘_i_*	Permeability of gas *i* (Barrer)
*p*	Partial pressure (bar)
*PM*	Permeance (GPU)
K	Dimensionless equilibrium constant
*k*	Reaction rate constant (s^−1^)
*J_i_*	Permeate flowrate of gas *i* (cm^3^ (STP)·s^−1^)
*D_AC_*	Diffusion coefficient of the carrier-permeant complex (cm^−2^∙s^−1^)
*p′*	upstream or feed partial pressure (bar)
*p″*	downstream partial pressure (bar)
*S*	Effective membrane surface area (cm^2^)
*C_T_*	Total carrier concentration (mol·cm^−3^)
*k_d,,CO2_*	Sorption coefficient of CO_2_ in the membranes (mol·cm^−3^∙bar^−1^)
*C*	Molar concentration (moL·cm^−3^)
z	distance from the upstream side of the membrane (m)
**Greek symbols**	
*α_m_*	Mobility ratio (dimensionless)
*α*	Membrane selectivity (dimensionless)
*ε*	Inverse Damkhöler number (dimensionless)
*K*	Reaction equilibrium number (dimensionless)
*α_i/j_*	CO_2_/N_2_ ideal selectivity between tow gas species *i* and *j* (dimensionless)
λ	A measure of the facilitation factor (dimensionless)
*℘_i_*	Permeability of gas *i* (Barrer)
**Subscripts**	
*f*	Feed
*A*	Solute
AC	Carrier-solute complex
r	Reverse
f	Forward
*i*	Compound
0	Upstream side (z = 0)
**Superscripts**	
*SD*	Relative to solution-diffusion
*	Relative to dimensionless
